# Restoration of miR-29b exerts anti-cancer effects on glioblastoma

**DOI:** 10.1186/s12935-017-0476-9

**Published:** 2017-11-17

**Authors:** Jaekyung Shin, Hyun Geun Shim, Taeyoung Hwang, Hyungsin Kim, Shin-Hyuk Kang, Yun-Sik Dho, Sung-Hye Park, Sang Jeong Kim, Chul-Kee Park

**Affiliations:** 1 Department of Neuroscience, Karolinska Institutet, Stockholm, Sweden; 2 Department of Physiology, Seoul National University College of Medicine, Seoul, South Korea; 3 Department of Biomedical Engineering, Johns Hopkins University School of Medicine, Baltimore, MD USA; 4 Department of Neurosurgery, Korea University College of Medicine, Seoul, South Korea; 5 Department of Neurosurgery, Seoul National University College of Medicine, Seoul National University Hospital, Seoul, South Korea; 6 Department of Pathology, Seoul National University College of Medicine, Seoul National University Hospital, Seoul, South Korea

**Keywords:** Glioblastoma, miR-29b, Anti-cancer effect, Nanoparticle

## Abstract

**Background:**

Glioblastoma multiforme (GBM) is known as one of the most fatal forms of cancer. MicroRNAs have been widely implicated in the regulation of mammalian development and pathogenesis. The brain-enriched miR-29 subfamilies are known to be exclusively expressed in the developing brain, and they are aberrantly down-regulated in GBM. This study aims to elucidate the role of miR-29b in GBM development and the feasibility of therapeutic targeting using conjugated nanoparticles.

**Methods:**

After confirmation of miR-29b expression levels in GBM tissues by analysis of open source data, the anticancer effect of miR-29b was tested by the introduction of syn-hsa-miR-29b-3p in the A172 GBM cell line. In vitro studies of cell viability and apoptosis and ex vivo study using GBM tissue slice cultures from 3 patients and nanoparticle delivery of miR-29b were performed.

**Results:**

We discovered an increase in apoptotic cell populations with the introduction of miR-29b in the GBM cell line. An established human-derived GBM tissue slice culture system confirmed the anticancer effect of miR-29b-conjugated nanoparticles. Using PCR array, we found that exogenous miR-29b inhibits the expression of COL1A2, COL3A1, COL4A1, ELN, ITGA11, MMP24, and SPARC, which mediates an anticancer effect.

**Conclusions:**

miR-29b may serve as a putative therapeutic molecule when its expression is restored using a nanoparticle delivery system in GBM.

**Electronic supplementary material:**

The online version of this article (10.1186/s12935-017-0476-9) contains supplementary material, which is available to authorized users.

## Background

Glioblastoma multiforme (GBM) is known as one of the most fatal forms of brain cancer in humans, with an average survival duration of approximately 14 months [1]. However, because of pacesetting and intensive research on genomic profiles in GBM, a substantial amount of knowledge regarding its oncogenesis is rapidly accumulating [2]. The current understanding of the genomic characteristics of GBM extend from the genetic and epigenetic characteristics of the disease to their regulatory mechanisms encompassing posttranslational modifications, chromosome remodelling, and small and long non-coding RNAs (ncRNAs).

MicroRNAs (miRNAs) are small, ncRNAs of approximately 22 nucleotides in length that can regulate the expression of messenger RNAs (mRNAs) [3]. The targets of a single miRNA can be multiple and multiple miRNAs can be used against a single target [4]. Accumulating evidence has demonstrated that miRNAs act as crucial regulators of cancer [5]. Since the initial studies that demonstrated the aberrant expression of miRNAs in GBM samples, hundreds of papers have been published regarding the role of miRNAs in GBM [6–8]. An extensive review of all those studies concluded that 253 miRNAs are significantly up-regulated and 95 miRNAs are down-regulated in GBM tissues compared with those in normal brain tissues [8]. However, most of the aberrantly expressed miRNAs are yet to be functionally characterized [8].

Based on our previous study, we investigated miR-29b for its function in inhibiting the self-renewal and proliferation of neural stem cells, which can significantly affect neurogenesis [9]. Therefore, we postulated that there might be a certain role of miR-29b in brain tumour development considering the similarity of tumour progenitor cells with neural stem cells. In humans, the genetic loci encoding miR-29 consist of two gene clusters, the miR-29a/b-1 locus on chromosome 7q32 and the miR-29b-2/c locus on 1q23. While miR-29a localizes to the cytoplasm, miR-29b and miR-29c are found in the nucleus [10, 11]. There is evidence of the role of miR-29s in a variety of cancer types [12]. Here, we demonstrate that miR-29b is a tumour suppressor that is deregulated in GBM, and its restoration can exert an anti-cancer effect.

## Methods

### Cell line and culture

The human GBM cell line A172 was purchased from American Type Culture Collection (ATCC^®^ CRL-1620™, Manassas, VA, USA) and cultured in Dulbecco’s Modified Eagle’s Medium (DMEM, WelGene, Dae-Ku, Korea) containing 10% foetal bovine serum (FBS, Gibco, Grand Island, NY, USA) and 5% antibiotics (streptomycin) in a humidified atmosphere of 5% CO_2_ and 95% air at 37 °C.

### RNA interference and reagents

For RNA interference, non-targeting control siRNA (#D-001610-01-05, Dharmacon, Lafayette, CO, USA) and syn-hsa-miR-29b-3p (#MSY0000100, Qiagen, Hilden, Germany) were purchased, and Lipofectamine^®^ RNAiMAX Reagent (#13776-150, Invitrogen, Carlsbad, CA, USA) was used to transfect cells.

### Flow cytometry

A total of 1 × 10^5^ A172 cells were plated in 6-well dishes and incubated with 30–50 nM control siRNA or syn-hsa-miR-29b-3p with Lipofectamine^®^ RNAiMAX Reagent according to the manufacturer’s protocol for 3 days at 37 °C. The cells were fixed with ice-cold 70% ethanol after harvesting, washed once with phosphate-buffered saline (PBS) and resuspended in PBS, and then ribonuclease A (0.1%) and propidium iodide (PI, 10 µg/ml) were added to the cells. The cells were incubated for 30 min in the dark at room temperature. For apoptosis assays, cells were harvested and suspended in Annexin V binding buffer (#556570, BD Biosciences Pharmingen, San Diego, CA, USA) at a density of 1 × 10^5^ cells/100 μl. Next, 5 μl of Annexin V-FITC and 5 μl of PI were added to each sample, and the samples were incubated in the dark at room temperature for 15 min.

Fluorescence events were quantified after laser excitation of the fluorescent dye with a fluorescence-activated cell sorter (FACSCalibur, Becton–Dickinson, Heidelberg, Germany) with a cell count of 1 × 10^5^–10^6^ cells per 0.2–0.3 ml. The data were processed using Cell Quest Pro software (version 5.2.1, BD Biosciences).

### Cell viability

Cell viability was tested using the LIVE/DEAD^®^ Viability/Cytotoxicity Kit (#L3224, Molecular Probes, Inc., Eugene, OR, USA) according to the manufacturer’s instructions. In brief, 5 × 10^4^ A172 cells were seeded onto a 0.1% gelatine-coated slides, and 20 μM syn-hsa-miR-29b-3p was added to a final concentration of 30–50 nM. Calcein-AM and ethidium bromide were added for 15 min at room temperature. The slides were mounted with 1× PBS and immediately photographed.

### Glioblastoma-derived tissue slice culture

Glioblastoma samples were obtained from 3 patients during surgery after the patients provided informed consent and the samples were immersed in ice-cold sterile 1× HBSS, orientated, mounted, and immobilized using cyanoacrylate glue immediately after resection. Using a Vibratome VT1200 (Leica Microsystems Inc., Buffalo Grove, IL, USA), the most viable regions of the tissue were selected, and 400 μm-thick slices were cut. The vibration amplitude was set at 2.0 mm, and the slicing speed was 0.03–0.05 mm/s. The tissue slices were placed in 6-well plates on membrane inserts with 0.4 μm pores (#PICM03050, Millipore Corp., Bedford, MA, USA). The tissue slices were cultured at 37 °C in a humidified incubator with 5% CO_2_ for 10 days using 1 ml of MEM supplemented with 10% FBS. The genetic characteristics of the samples used for the tissue slice culture are summarized in Additional file 1: Table S1.

### Nanoparticle complex

We used InViVojection™ RNAi-nano Red reagent (#DHMSN-vivoRFRNA25, Lemonex Inc., Seoul, Korea) for the delivery of control siRNA and miR-29b into tissue slice cultures according to the manufacturer’s instructions. In brief, 80 μl of InViVojection™ reagent was mixed with 10 μl of 10× PBS in the dark, and 10 μl of miR-29b (100 μM stock) or control siRNA was added. After incubation at room temperature for 1 h, the reagent mixture was carefully dropped on top of the slices to soak in. The incorporation of nanoparticle complex into tissues was confirmed by transmission electron microscopy (TEM).

### Immunohistochemistry

The cultured tissue slices at day 0 and at day 7 were formalin-fixed and paraffin-embedded (FFPE) and cut into 4 μm sections for immunohistochemistry (IHC).

To assay proliferation, primary antibodies against human Ki-67 (1:1000, #M724029, DAKO, Glostrup, Denmark) were used. A nuclear algorithm of the Aperio ScanScope image analysis program (Aperio Technologies, Vista, CA, USA) was used to calculate the nuclear positivity in hot spots for the Ki-67 labelling index.

To assay apoptosis, terminal transferase was used to visualize fragmented DNA (terminal deoxynucleotidyl transferase-mediated dUTP nick-end labelling, TUNEL labelling, In Situ Cell Death Detection Kit, POD, #1,684,817, Roche Applied Science, Mannheim, Germany). The apoptotic index (%) was calculated by dividing the number of apoptotic nuclei by the total number of nuclei counted. The cells were counterstained with methyl green.

### Polymerase chain reaction array

To identify the targets of human miR-29b in GBM cells, we performed polymerase chain reaction (PCR) using the RT^2^ Profiler™ PCR Array kit (#PAHS-6012Z, SA Biosciences, Frederick, MD, USA). C_t_ values were converted to fold change, using web-accessible analysis programs (http://pcrdataanalysis.sabiosciences.com/pcr/arrayanalysis.php). Fold change ($2^{{ - \Delta \Delta {\text{C}}_{\text{T}} }}$) was calculated from the normalized gene expression ($2^{{ - \Delta {\text{C}}_{\text{T}} }}$) in the test sample divided by the normalized gene expression ($2^{{ - \Delta {\text{C}}_{\text{T}} }}$) in the control sample. We present the target molecules with their log_2_ fold change (x-axis) and the corresponding log_2_ *p* values (y-axis) on a volcano plot.

### Public database usage and statistical analysis

To screen the miR-29b expression levels in GBM tissues, we curated and combined the open source data from two previous studies comparing GBM tissues (n = 9) and non-tumour brain tissues (n = 6) using miRNA-seq [13, 14]. Specifically, raw counts of the sequencing reads for commonly identified miRNAs in both studies were obtained for every sample using the supplementary data from the studies. The differential expression of miRNAs between glioblastoma and non-tumour brain tissues was statistically evaluated using the R package DESeq2 that uses a negative binomial model [15]. In particular, we controlled for batch effect arising from the integration of the two separate studies by considering a variable that identifies the study providing the samples being analysed.

An integrated network analysis of the gene of interest was conducted and visualized using PCViz in Pathway Commons (http://www.pathwaycommons.org) [16].

Quantitative data were expressed as the mean ± the standard error of the mean (SEM). The statistical significance between two groups was tested using the two-tailed Student’s *t* test. A p value of < 0.05 was considered significant.

## Results

### Expression of miR-29b is suppressed in GBM

**Fig. 1:**
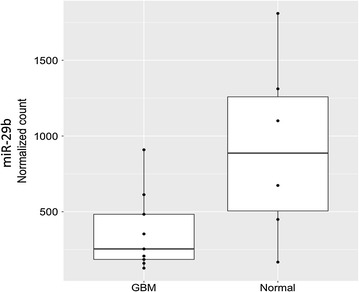
Comparison of miR-29b expression levels between glioblastoma (GBM, n = 9) and normal brain tissues (Normal, n = 6) showing significant down-regulation of miR-29b in GBM (p = 0.02). A normalized count of miR-29b from open source miRNA-seq data was used [13, 14]

We used open source miRNA profiling data from 2 previously published studies to compare the levels of expression of miR-29b between GBM and normal brain tissues [13, 14]. After correction for the batch effect of each dataset, a total of 9 GBM and 6 normal brain tissues could be analysed for 423 miRNAs that were commonly expressed. Among those miRNAs, a total of 170 miRNAs were significantly differentially expressed between the GBM and normal brain tissues (Table 1; Additional file 1: Tables S2, FDR < 0.1). Based on our previous study, we focused on miR-29b for its function in inhibiting the self-renewal and proliferation of neural stem cells, which can significantly affect neurogenesis [9]. miR-29b was found to be significantly down-regulated in GBM (Fig. 1, log_2_ (Normal/GBM) = 1.34, p = 0.02, FDR = 0.06).

**Table 1:** A total of 76 differentially expressed microRNAs with significantly lower expression in glioblastoma (GBM) compared to non-tumour brain tissues

No.	MicroRNA	log2(FC)	log2(FC-standard error)	z-statistic	p value	FDR
1	hsa-miR-124	7.5068	1.0387	7.2270	0.0000	0.0000
2	hsa-miR-218	6.6782	0.9775	6.8322	0.0000	0.0000
3	hsa-miR-873	5.4139	1.0405	5.2031	0.0000	0.0000
4	hsa-miR-7	4.7696	0.7774	6.1350	0.0000	0.0000
5	hsa-miR-1912	4.5089	1.8598	2.4243	0.0153	0.0429
6	hsa-miR-129star	4.5065	0.7294	6.1782	0.0000	0.0000
7	hsa-miR-139-3p	4.3718	0.8802	4.9666	0.0000	0.0000
8	hsa-miR-383	4.1483	1.0614	3.9083	0.0001	0.0007
9	hsa-miR-495	4.0638	0.9937	4.0894	0.0000	0.0004
10	hsa-miR-139-5p	3.8873	0.9888	3.9314	0.0001	0.0007
11	hsa-miR-138	3.7940	0.8260	4.5930	0.0000	0.0001
12	hsa-miR-485-5p	3.6911	0.7200	5.1262	0.0000	0.0000
13	hsa-miR-323-3p	3.5294	0.6566	5.3754	0.0000	0.0000
14	hsa-miR-487b	3.5207	0.7118	4.9464	0.0000	0.0000
15	hsa-miR-485-3p	3.4738	0.7005	4.9591	0.0000	0.0000
16	hsa-miR-1286	3.4257	0.9717	3.5256	0.0004	0.0025
17	hsa-miR-1224-5p	3.3436	0.7768	4.3045	0.0000	0.0002
18	hsa-miR-132	3.3423	0.6392	5.2287	0.0000	0.0000
19	hsa-miR-543	3.2264	0.6293	5.1272	0.0000	0.0000
20	hsa-miR-490-3p	3.1761	0.7582	4.1889	0.0000	0.0003
21	hsa-miR-490-5p	3.1672	0.8236	3.8454	0.0001	0.0009
22	hsa-miR-410	3.0772	0.8013	3.8405	0.0001	0.0009
23	hsa-miR-668	3.0100	0.7603	3.9588	0.0001	0.0006
24	hsa-miR-323b-3p	2.9812	0.8378	3.5582	0.0004	0.0023
25	hsa-miR-212	2.8464	0.6536	4.3552	0.0000	0.0002
26	hsa-miR-628-5p	2.8262	0.5173	5.4638	0.0000	0.0000
27	hsa-miR-124star	2.8005	0.8573	3.2666	0.0011	0.0055
28	hsa-miR-136	2.7895	0.7544	3.6977	0.0002	0.0014
29	hsa-miR-935	2.7497	0.7178	3.8304	0.0001	0.0009
30	hsa-miR-539	2.7319	0.9807	2.7857	0.0053	0.0194
31	hsa-miR-504	2.7238	0.8304	3.2800	0.0010	0.0053
32	hsa-miR-487a	2.7167	0.7557	3.5948	0.0003	0.0021
33	hsa-miR-138-1star	2.6958	0.5442	4.9533	0.0000	0.0000
34	hsa-miR-132star	2.6106	0.8890	2.9365	0.0033	0.0145
35	hsa-miR-770-5p	2.5856	0.9006	2.8711	0.0041	0.0165
36	hsa-miR-433	2.5657	1.0518	2.4394	0.0147	0.0415
37	hsa-miR-4446-3p	2.5107	0.9984	2.5147	0.0119	0.0351
38	hsa-miR-154star	2.5037	0.6443	3.8859	0.0001	0.0008
39	hsa-miR-375	2.4955	0.7418	3.3642	0.0008	0.0042
40	hsa-miR-431star	2.4278	0.8736	2.7791	0.0055	0.0196
41	hsa-miR-411star	2.3678	0.5828	4.0629	0.0000	0.0004
42	hsa-miR-342-3p	2.3513	0.6636	3.5430	0.0004	0.0024
43	hsa-miR-376astar	2.3385	0.8987	2.6022	0.0093	0.0293
44	hsa-miR-431	2.3223	1.1063	2.0991	0.0358	0.0851
45	hsa-miR-496	2.2728	0.8521	2.6672	0.0076	0.0254
46	hsa-miR-7-1star	2.1583	0.8207	2.6299	0.0085	0.0274
47	hsa-miR-769-3p	2.1347	0.5331	4.0047	0.0001	0.0005
48	hsa-miR-655	2.0954	0.9282	2.2574	0.0240	0.0606
49	hsa-miR-432	2.0845	0.7328	2.8443	0.0045	0.0175
50	hsa-miR-203	2.0536	0.7244	2.8349	0.0046	0.0175
51	hsa-miR-382	2.0529	0.7358	2.7901	0.0053	0.0193
52	hsa-miR-329	2.0327	0.8035	2.5298	0.0114	0.0344
53	hsa-miR-491-5p	2.0173	0.7588	2.6586	0.0078	0.0257
54	hsa-miR-221	1.9337	0.6314	3.0623	0.0022	0.0102
55	hsa-miR-379	1.9324	0.7444	2.5961	0.0094	0.0296
56	hsa-miR-369-5p	1.8083	0.7021	2.5755	0.0100	0.0307
57	hsa-miR-330-3p	1.8050	0.6517	2.7699	0.0056	0.0199
58	hsa-miR-379star	1.7531	0.7448	2.3540	0.0186	0.0502
59	hsa-miR-149	1.7529	0.6027	2.9083	0.0036	0.0153
60	hsa-miR-1	1.7498	0.4676	3.7418	0.0002	0.0012
61	hsa-miR-889	1.7203	0.7117	2.4174	0.0156	0.0435
62	hsa-miR-136star	1.6970	0.6581	2.5789	0.0099	0.0306
63	hsa-miR-153	1.5730	0.6368	2.4703	0.0135	0.0392
64	hsa-miR-766	1.5411	0.6396	2.4093	0.0160	0.0438
65	hsa-miR-377star	1.4851	0.6731	2.2063	0.0274	0.0678
66	hsa-miR-411	1.4768	0.6314	2.3389	0.0193	0.0513
67	hsa-miR-377	1.4701	0.6668	2.2046	0.0275	0.0678
68	hsa-miR-95	1.4609	0.6567	2.2247	0.0261	0.0656
69	hsa-miR-376a	1.4324	0.7042	2.0342	0.0419	0.0972
70	hsa-miR-127-5p	1.3517	0.6337	2.1329	0.0329	0.0787
71	hsa-miR-29b	1.3389	0.5925	2.2597	0.0238	0.0606
72	hsa-miR-425	1.3189	0.6458	2.0421	0.0411	0.0960
73	hsa-let-7d	1.2599	0.5884	2.1411	0.0323	0.0776
74	hsa-miR-191	1.1826	0.4351	2.7181	0.0066	0.0222
75	hsa-miR-598	1.1699	0.5339	2.1911	0.0284	0.0697
76	hsa-miR-769-5p	1.1113	0.4806	2.3121	0.0208	0.0543

Whole set of differentially expressed microRNAs between GBM and non-tumor brain tissues are listed in Additional file 1: Table S2. A total of 170 microRNAs were found to be significant after combining open source data from two previous studies comparing GBM (n = 9) and non-tumour brain tissues (n = 6) with miRNA-seq [13, 14]. Log2(FC) indicates log2(fold change of non-tumour brain tissues to GBM)

### Restoration of miR-29b induces cell death and growth arrest in a GBM cell line

**Fig. 2:**
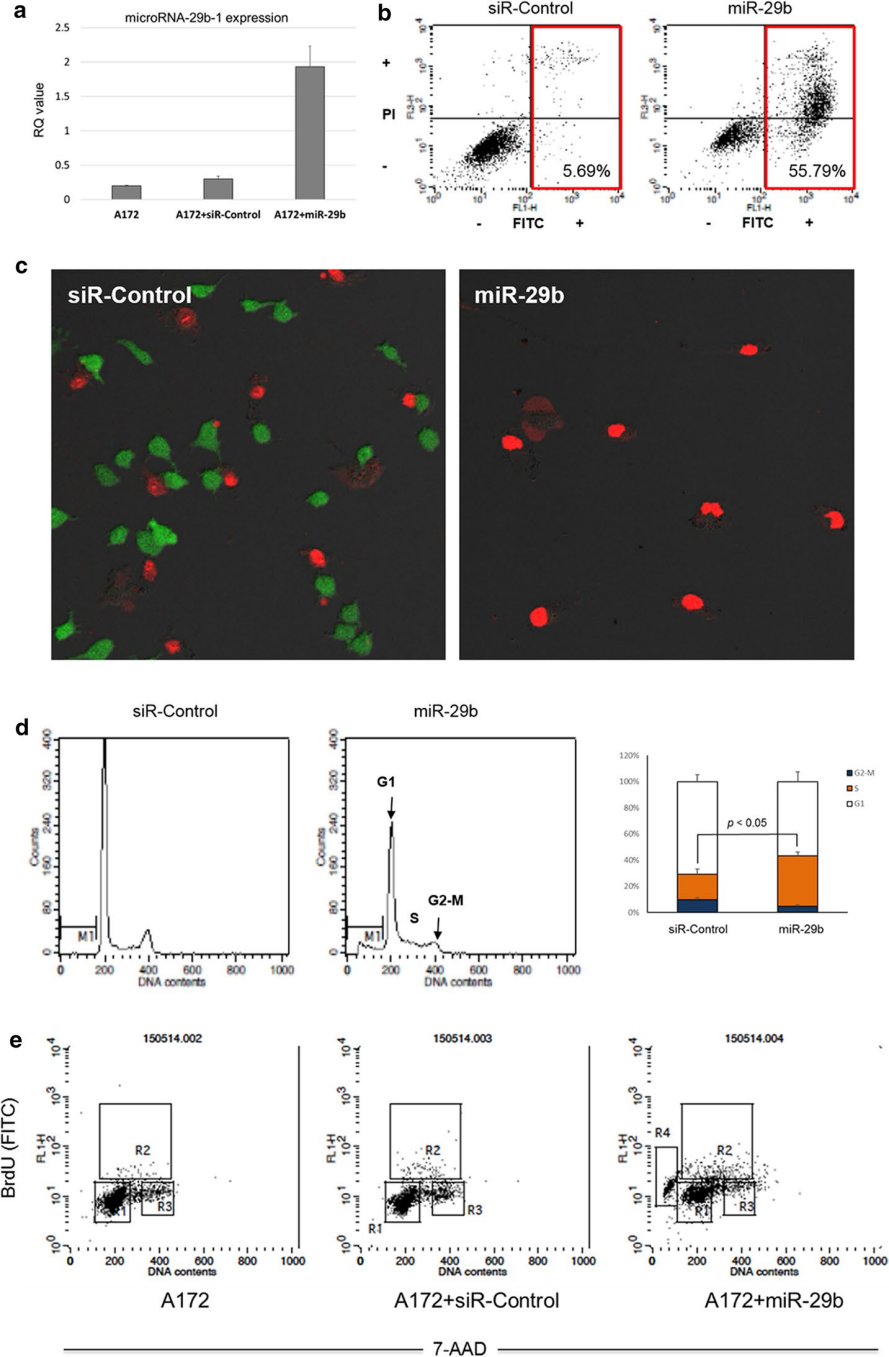
Anticancer effects of miR-29b on the A172 cell line. **a** Endogenous expression of miR-29b in A172, which is successfully overexpressed after transfection of miR-29b. **b** Restoration of miR-29b induces apoptosis in more than 50% of cells as shown by FACS analysis with Annexin V staining. **c** LIVE/DEAD assay showing a decrease in live cells after miR-29b treatment. **d** Cell cycle analysis by quantitation of the DNA content showing significant S phase arrest (p < 0.05) in miR-29b-treated cells. Error bars indicate the mean ± SEM for n > 3. Significance was determined using Student’s t test. **e** Effects of miR-29b on cell cycle progression were studied by flow cytometry using the FITC-BrdU/7-AAD assay. Expression of miR-29b significantly induced apoptosis (R4, 19.6%), and S-phase cell cycle arrest (R2, 12.82%) in A172 cells. Insignificant changes were observed in fractions representing G1-phase (R1), and G2/M-phase (R3)

Introduction of miR-29b into A172, a PTEN-deficient GBM cell line, showed successful overexpression (Fig. 2a). FACS analysis with Annexin V staining revealed a marked increase in the apoptotic cell fraction after miR-29b treatment (Fig. 2b). The LIVE/DEAD cell staining confirmed a decrease in the viability of the miR-29b-treated cells (Fig. 2c). Cell cycle analysis showed a significant S-phase arrest after miR-29b treatment (Fig. 2d). Overall, miR-29b acts as a tumour suppressor, which can inhibit cell growth and induce apoptosis in vitro. To confirm the growth arrest effect of miR-29b, we performed flow cytometry experiments using the BrdU/7-AAD assay. Expression of miR-29b was followed by a significant induction of apoptosis and S-phase arrest in A172 cells, while the fraction of G1 and G2/M phase remained unaltered (Fig. 2e).

### Introduction of nanoparticle loaded with miR-29b exerts anti-cancer effects on GBM-derived tissues in culture

**Fig. 3:**
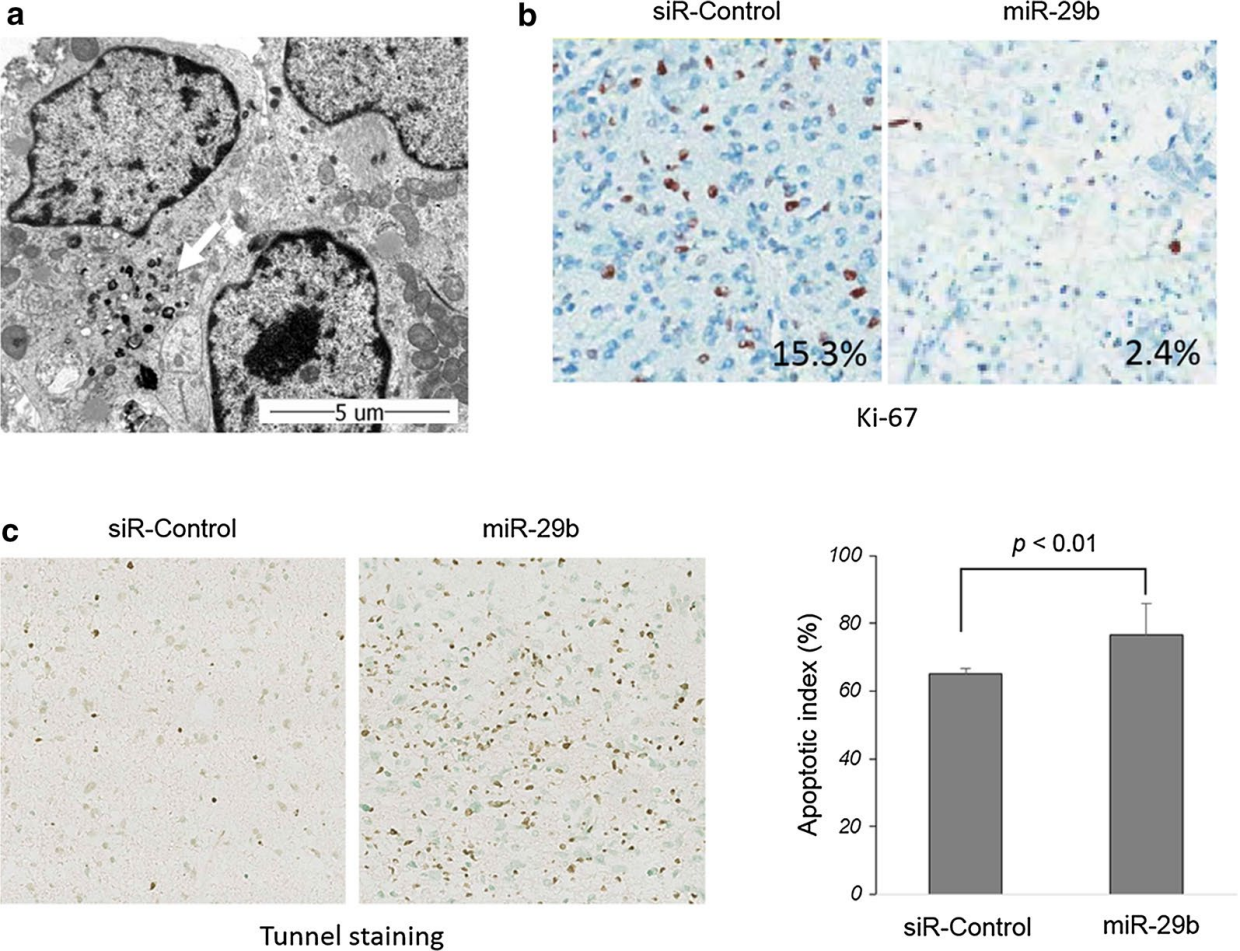
Anticancer effects of miR-29b on glioblastoma tissue slice cultures. **a** Transmission electron microscopy (TEM) images of cultured tissues showing incorporation of nanoparticles loaded with miR-29b (arrow). **b** Ki-67 immunostaining on the glioblastoma tissues cultured for 7 days after miR-29b treatment shows a decrease in the proliferative index (2.4%) compared with control siRNA treatment (15.3%). **c** The apoptosis rate was measured by quantifying the number of apoptotic cells positive for TUNEL. A significant increase in the apoptotic index is observed after miR-29b treatment compared with control siRNA treatment (p < 0.01). Error bars represent the mean ± SEM for n > 3. Significance was determined using Student’s t test

We cultured GBM tissue slices (n = 3) for testing the anticancer effects of miR-29b ex vivo. The delivery of miR-29b into cancer cells in cultured tissue could be effectively achieved using nanoparticles (Fig. 3a). The anticancer effects were evaluated 7 days after treatment. There was a marked decrease in proliferation and significant increase in apoptosis after miR-29b treatment (Fig. 3b, c).

### Targets of miR-29b in GBM cells

**Fig. 4:**
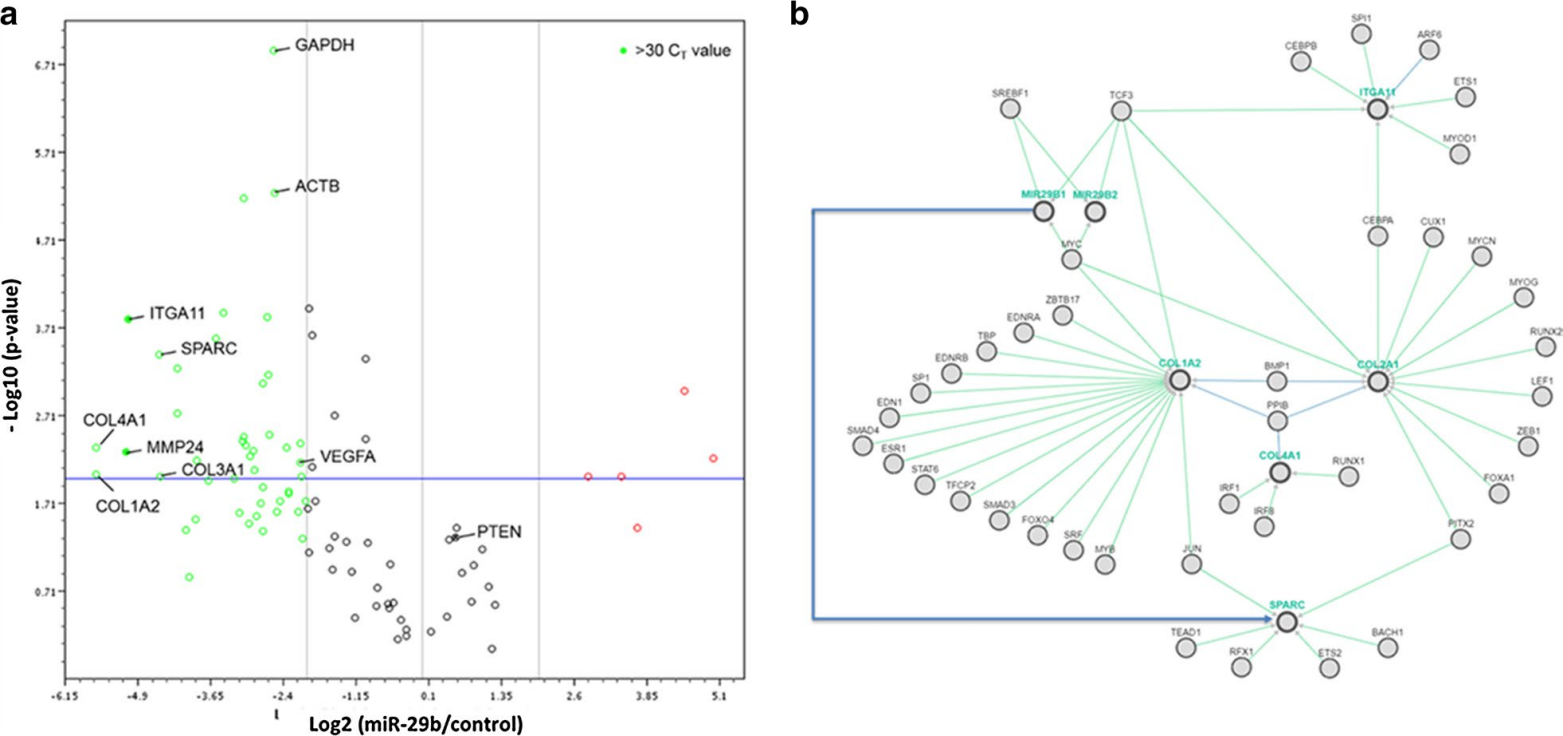
Mediators of the effects of miR-29b on glioblastoma cells. **a** Volcano plots showing miR-29b targeted genes analysed with a PCR array using naïve- and miR-29b-treated A172 cells. The significant candidate targets (fold change > 20) include COL1A2, COL4A1, COL2A1, ITGA11, MMP24, and SPARC. **b** Network analysis incorporating genes of interest using Pathway Commons (http://www.pathwaycommons.org) shows relationships among the genes. Direct inhibition of SPARC by miR-29b is annotated with a solid line

To elucidate miR-29b target genes in GBM, we analysed A172 cells using the Human miR-29 Targets RT^2^ Profiler PCR Array (QIAGEN #PAHS-6012Z) (Additional file 1: Table S3). By comparing the expression levels of 96 candidate target genes before and after miR-29b treatment, we found 75 genes that showed significant changes (p < 0.05) of either increased expression (10 genes) or decreased expression (65 genes). The noticeably down-regulated genes were COL1A2, COL4A1, COL2A1, ITGA11, MMP24, and SPARC (Fig. 4a). A network was constructed based on the regulatory relationships of the genes of interest using Pathway Commons (Fig. 4b). Among them, SPARC was found to be of particular interest, as its expression was remarkably reduced (21.14-fold decreased) after miR-29b treatment from being highly expressed in the GBM cell line.

## Discussion

Similar to other cancer types, it has been theorized that GBM arises from neural stem cells (NSCs) that undergo genetic mutations in tumour suppressor genes and oncogenes [17]. Non-neoplastic cells that transform and produce gliomas are likely the direct descendants of common NSCs [18]. Therefore, it is plausible that oncogenesis of GBM shares a common genetic developmental mechanism with NSCs. We have previously shown that miR-29b plays a pivotal role in neurogenesis by regulating the inhibitor of β-catenin and T cell factor (ICAT)-mediated Wnt/β-catenin signalling in NSCs, which impacts their self-renewal and proliferation [9]. MicroRNAs regulate a wide range of gene expression in a post-transcriptional manner, and their aberrant function affects a variety of steps in tumour development. Among the microRNAs that are known to be deregulated in GBMs, miR-29b attenuates stemness in NSCs during brain development [9]. In the present study, we showed that the restoration of down-regulated miR-29b in GBM cells exerts anticancer effects by inhibiting cell cycle arrest. Furthermore, we demonstrated that the loss of ECM molecules in A172 cells accelerates cell apoptosis in GBMs. These results can be mediated via the epithelial-mesenchymal transition (EMT)-related processes that are correlated with ECM molecules including COL1A2, COL3A1, COL4A1, ELN, ITGA11 and MMP24. Accumulating evidence has shown that the miR-29 family is involved in multiple cancer types [5]. Also, miR-29b is generally recognized as a fundamental regulator of EMT [5].

Among the possible functional mediators of miR-29b, SPARC is of particular interest, as its expression was remarkably reduced after miR-29b treatment in GBM. SPARC induces changes in the migration [19], invasion [20] and angiogenesis of GBM cells in vitro. Additionally, SPARC affects tumour cell behaviour to activate the PI3K (phosphoinositide-3-kinase)-AKT pathway, a key mediator of cell survival [21]. SPARC can act as a potent oncogene and therapeutic RNAi target in GBM. This is in line with a previous study on breast cancer cells showing that C1QTNF6, SPARC, and COL4A2 were targeted by miR-29b [22]. Another study showed that miR-29b exerts a critical suppressive role on colorectal cancer mediated by the inhibition of Tiam1 and EMT [23].

## Conclusions

miR-29b may be used as a therapeutic molecule when its expression is restored, which indicates its potential role in the molecular therapy of patients with GBM. Although the induction of apoptosis is the predominant anti-cancer role of miR-29b, analysis of the mediators of the miR-29b effect suggests that diverse mechanisms can contribute to tumour suppression, which need to be evaluated in the future.

## Additional file

**Additional file 1.** Supplementary tables.
